# A Comparison of Plain Radiography with Computer Tomography in Determining Coronal and Sagittal Alignments following Total Knee Arthroplasty

**DOI:** 10.5704/MOJ.1707.006

**Published:** 2017-07

**Authors:** GN Solayar, J Chinappa, IA Harris, DB Chen, SJ Macdessi

**Affiliations:** Department of Orthopaedics, International Medical University (IMU), Seremban, Malaysia; ^*^Department of Orthopaedics, Canterbury Hospital, Sydney, Australia; ^**^Ingham Institute for Applied Medical Research, South Western Sydney Clinical School, UNSW Australia, Sydney, Australia; ^***^Sydney Knee Specialists, St George Private Hospital, Sydney, Australia

**Keywords:** radiographs, computer tomography, coronal alignment, sagittal alignment, total knee arthroplasty

## Abstract

**Introduction:** Optimal coronal and sagittal component positioning is important in achieving a successful outcome following total knee arthroplasty (TKA). Modalities to determine post-operative alignment include plain radiography and computer tomography (CT) imaging. This study aims to determine the accuracy and reliability of plain radiographs in measuring coronal and sagittal alignment following TKA.

**Materials and Methods:** A prospective, consecutive study of 58 patients undergoing TKA was performed comparing alignment data from plain radiographs and CT imaging. Hip-knee-angle (HKA), sagittal femoral angle (SFA) and sagittal tibial angle (STA) measurements were taken by two observers from plain radiographs and compared with CT alignment. Intra- and inter-observer correlation was calculated for each measurement.

**Results:** Intra-observer correlation was excellent for HKA (r>0.89) with a mean difference of <1.9°. The least intra-observer correlation was for SFA (mean r=0.58) with a mean difference of 8°. Inter-observer correlation was better for HKA (r>0.95) and STA (r>0.8) compared to SFA (r=0.5). When comparing modalities (radiographs vs CT), HKA estimations for both observers showed the least maximum and mean differences while SFA observations were the least accurate.

**Conclusion:** Radiographic estimation of HKA showed excellent intra- and inter-observer correlation and corresponds well with CT imaging. However, radiographic estimation of sagittal plane alignment was less reliably measured and correlated less with CT imaging. Plain radiography was found to be inferior to CT for estimation of biplanar prosthetic alignment following TKA.

## Introduction

Establishing optimal mechanical alignment in total knee arthroplasty (TKA) surgery is a central component to achieving a successful outcome. It has been recommended that femoral and tibial components be positioned with less than 3° of error in the coronal plane to improve functional outcomes and avoid alignment associated complications^1^. Optimal sagittal positioning, according to some authors, include the femoral component positioned between 0 to 3° and the tibial component between 0 to 7°^2,3^. Post-operative alignment data is often obtained using long-leg radiographs. Post-operative radiographic measurements have been reported as reliable by some authors^4–6^ while other studies report significant variations^7–10^. Other methods of determining alignment such as CT^11^, MRI^12^, intra-operative supine fluoroscopy^8^ and intra-operative navigation^13–14^ have been reported previously with varying results. There is evidence to suggest equivocal sensitivities between plain radiography and CT imaging in estimating the post-operative mechanical axis in the coronal plane^4^.

Most research has been dedicated to coronal alignment following TKA; less emphasis has been placed on the accuracy of prosthetic alignment in the sagittal plane. Sagittal alignment may influence the outcome of TKA in different ways: failure of accurate sagittal plane component positioning may result in polyethylene post wear, femoral cortical notching, limitation of motion and possible fractures^15–16^. Determining sagittal component alignment using plain radiographs is challenging due to the effects of femoral bowing and difficulty in establishing a true sagittal axis using standard lateral radiographs of the knee that also include the hip and ankle joint. To the best of our knowledge, there are no studies correlating the accuracy of plain radiography versus CT in the sagittal plane.

The benefits of plain radiography include its availability, affordability, and the ability to detect the influence of weight-bearing on lower limb alignment. Limitations of radiographs are potential inaccuracy stemming from alterations in patient positioning and certain anatomic configurations (rotation and flexion contractures in particular) as described by some authors^17^–^18^. A full length lateral radiograph of the femur from hip to knee centres is difficult to obtain and as such a true sagittal mechanical axis determination is usually not possible. This measurement is often estimated from anatomic axis projections extending proximally from the knee joint for varying distances depending on the length of the x-ray plate. Plain radiographs are also unable to measure rotational alignment of the femoral and tibial implants.

Computer tomography may improve accuracy by negating the effects of patient position and improve detection of anatomic landmarks in all three planes (axial, coronal and sagittal). Full length mechanical axis determination is possible for both the femur and the tibia. Limitations of CT include the inability of incorporating weight-bearing and the higher cost. In addition, the effect of radiation exposure is a concern when using CT imaging. Radiation dose is generally quoted as the weighted dose (mSv) received by the body’s radiosensitive organs. The Perth protocol lower limb CT scans (which was used in this study) gives a dose of 2.7mSv^19^. In comparison, a standard long-leg standing radiograph gives a dose of about 0.7 mSv^11^. The average Australian background radiation was around 2 mSv per year^20^.

The objective of this study was to investigate the accuracy and reliability of plain radiographs in measuring coronal and sagittal alignment following TKA, using CT imaging as the gold standard. If measurements from plain radiographs could be shown to be accurate, CT tomography could then be limited to assessing rotational positioning of knee components. This could potentially result in a decrease in radiation exposure to the patient as well as reducing costs incurred for this imaging.

## Materials and Methods

We performed a prospective study of 62 consecutive patients who underwent TKA at our institution between December 2014 and April 2015. Four patients were excluded due to incomplete or insufficient radiographs. The remaining 58 patients were included in this study. Demographics of the study population are summarised in Table I. Pre-operative hip-knee angle (HKA) was measured in all patients. Postoperative CT imaging and plain radiographs were evaluated and analysed. This study was approved by the institutional ethics committee.

**Table I:** Demographics and pre-operative details

**Parameter**	**Study Group**
Age* (years)	66.4 ± 7.8 (47-88)
Involved knee (R/L)	32 / 27
Sex (F/M)	33 / 26
Alignment method (Navigated/IM)	14 / 44
Pre-op Hip-Knee Angle*	-2.65° ± 6.53° (-13° to 13°)**

^*^Values are shown as the mean and standard deviation with the range in parentheses.

^**^Positive values denote a valgus angle; Negative values denote varus angulature.

All surgeries were performed by two fellowship-trained orthopaedic surgeons. All patients received a cemented, posterior-stabilised, total condylar knee arthroplasty (Legion™, Smith & Nephew). A medial para-patellar approach was utilised and all operations were performed using a measured resection technique. We aimed for restoration of a neutral hip-knee angle in the coronal plane, 3 degrees of femoral component flexion in the sagittal plane and 3 degrees posterior tibial slope.

**Fig. 1:**
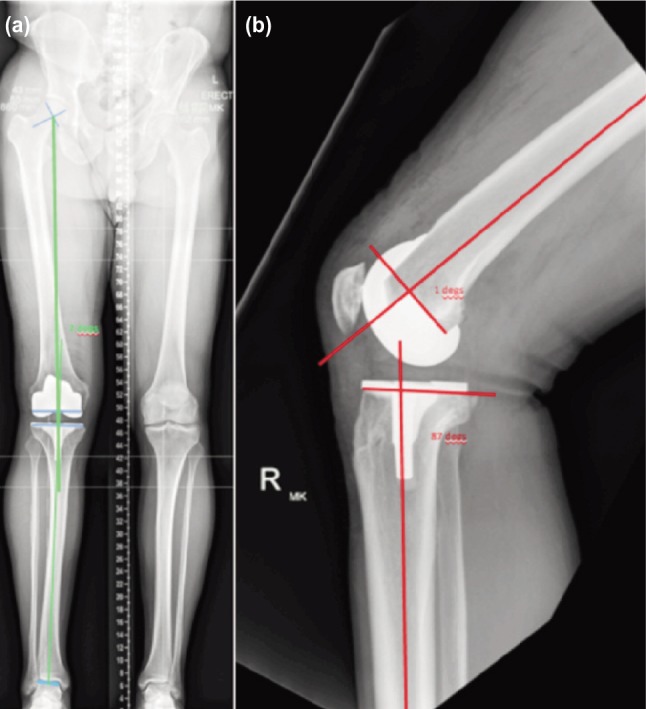
(a) Post-operative measurements of Hip-Knee Angle on 4-foot standing films and (b) Sagittal Femoral Angle and Sagittal Tibial Angle on 18-inch long lateral radiographs.

All patients in this series underwent post-operative plain radiographs using digital imaging. This included a 4-foot long standing film of the lower limb as well as an 18-inch, non-weight bearing lateral radiographs of the operated knee on the second day post-operatively (Fig. 1). The 4-foot films were used to assess coronal plane alignment and the 18-inch lateral film was chosen to optimise the chance of determining the true sagittal femoral and tibial mechanical alignment based on anatomic axis projections. For lateral radiographs, the patient’s foot was placed in a positioner which allowed for knee flexion between 30° to 45°. Limb rotation was deemed satisfactory if there was complete overlap of the medial and lateral femoral condyles on the lateral radiograph. Component alignments were evaluated, as described by Hsu *et al* ^21–22^ to determine, (1) the hip-knee angle (HKA) determined by the mechanical axes of both the femur and the tibia, (2) the sagittal femoral angle (SFA) determined between the sagittal anatomic distal femoral axis and the perpendicular axis of the femoral component and (3) the sagittal tibial angle (STA) between the sagittal anatomic proximal tibial axis and the perpendicular axis of the tibial component. Target surgical alignment values were a HKA of 0º in the coronal plane, a SFA of 3º for the femoral component and a STA of 3º of posterior slope for the tibial component.

In addition, all patients underwent a low dose CT scan using the Perth CT Protocol^19^. This technique allows non-weight bearing measurements of the femoral and tibial component alignments in the coronal, sagittal and axial plains with the added benefit of assessing rotational positioning. The anatomical landmarks determined included the centre of the femoral head, the centre of the talus, mid-point of the intercondylar notch and centre of the polyethylene insert or tibial plateau.

**Fig. 2:**
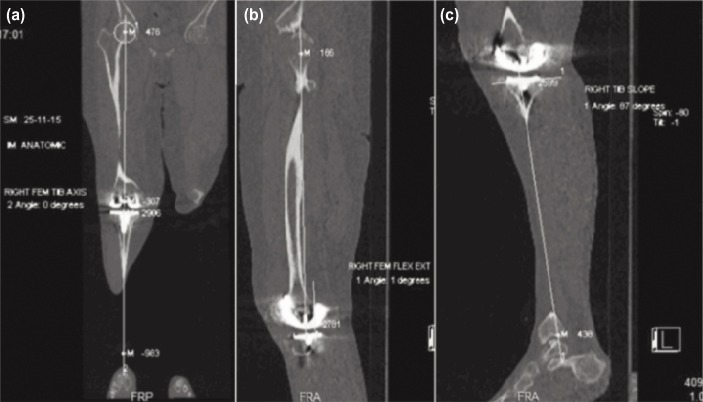
Post-operative measurements of (a) Hip-Knee Angle, (b) Sagittal Femoral Angle and (c) Sagittal Tibial Angle on CT radiographs using the CT Perth Protocol.

The CT tube current was set at 120 kV with an effective mAs of 50-70. Helical scans covered from just above the acetabulum to just below the ankle joint with image reconstructions of 2mm thickness at 1.6mm intervals. The limb was positioned with the knee in full extension and rotated in to the AP position. All measurements were performed on a Siemens workstation. Selected images were then chosen from the 3D tab and then linked to mark the centre of each region on all images. This technique allowed accurate reformatting of the limb in the true frontal, sagittal and axial planes so that rotational positional errors were minimised. Our institutions chief radiographer performed all CT scans and undertook measurements of HKA, SFA and STA (Fig. 2).

All standard radiographic measurements were independently performed by two observers, one an orthopaedic surgeon (GS) and the other an orthopaedic resident (JC). CT radiographic measurements were performed by the chief radiographer (NT) at our institution with CT Perth Protocol measurement experience over 5 years. The *syngo* CT2012a® software (Siemens Healthcare GmbH) was utilised for all radiographic and CT measurements.

Statistical analyses were performed using Microsoft Excel 2013 (Microsoft Corp., Redmond, WA, USA) software and MedCalc® version 15.4 (© 1993-2015 MedCalc Software bvba, Ostend, Belgium). The Pearson Product Moment Correlation test was used to determine intra- and inter-observer alignment measurements of post-operative radiographs (HKA, SFA and STA). Bland-Altman plots were utilised to assess agreement and are represented graphically. The 95% limits of agreement between observers are defined as the mean difference plus and minus 1.96 times the standard deviation of the differences^23^. Coefficient of repeatability was defined as two standard deviations of the difference between measurement and was a good indication of repeatability.

## Results

When measurements obtained by each of the observers were compared, an excellent correlation was found for each observer looking at HKA using long-leg radiographs (r=0.92 and r=0.89) with a mean difference of -0.03° (range -1.74 to 1.66°) and -0.16° (range -1.96 to 1.65°) for each observer. It was noted that the correlation reduced for both observers when measuring for SFA (r=0.67 and r=0.51) and STA (r=0.75 and r=0.47). The mean difference for measuring SFA was -0.07° (range -3.79 to 3.66°) and -2.04° (range -8.03 to 3.94°) for each observer while the mean difference for STA was 0.21° (range -2.76 to 3.2°) and -0.72° (range -4.72 to 3.29°) (Table II).

**Table II:** Intra-observer correlation by radiographic measurement

	**Hip-Knee Angle**			**Sagittal Femoral Angle**			**Sagittal Tibial Angle**		
	**Maximum difference**	**Mean difference**	SD*	**Maximum difference**	**Mean difference**	SD*	**Maximum difference**	**Mean difference**	SD*
Observer 1	3.2°	0.66°	0.56	6°	1.47°	1.16	3.5°	1.21°	0.94
Observer 2	2.6°	0.71°	0.6	12.1°	2.61°	2.58	6.4°	1.69°	1.34

^*^Standard Deviation

**Fig. 3:**
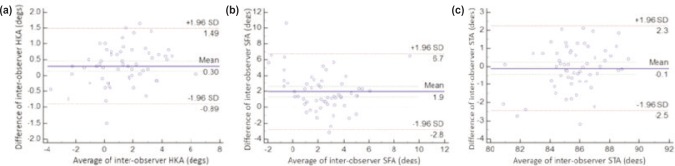
Relationship between the difference and mean of inter-observer radiographic measurements for (a) Hip-Knee Angle, (b) Sagittal Femoral Angle and (c) Sagittal Tibial Angle.

When measuring for HKA, an excellent inter-observer correlation was found with r=0.96 (range 0.93-0.97, p<0.0001) with a mean difference of 0.3°. The correlation figures were lower for STA (r=0.81, range 0.7 to 0.88, p<0.0001) and the mean difference was -0.1°. Measuring SFA provided the worst inter-observer correlation SFA with r=0.5 (range 0.28-0.67, p=0.0001) and a mean difference of 1.95° (Table III). Graphical representation to indicate agreement for inter-observer measurements are provided in Figure 3.

**Table III:** Inter-observer correlation by radiographic measurements

**Hip-Knee Angle Mean**			**Sagittal Femoral Angle**			**Sagittal Tibial Angle**		
**Maximum difference**	**Mean difference**	SD*	**Maximum difference**	**Mean difference**	SD*	**Maximum difference**	**Mean difference**	SD*
1.65°	0.54°	0.4	10.7°	2.37°	2.02	3.2°	0.93°	0.76

^*^Standard Deviation

For Observer 1, the correlation between radiographic HKA, SFA and STA measurements compared to CT results were r=0.75 (p<0.0001), r=0.18 (p=0.19) and r=0.66 (p<0.0001) respectively. For Observer 2, the HKA, SFA and STA correlations were r=0.79 (p<0.0001), r=-0.10 (p=0.44) and r=0.66 (p<0.0001) respectively. (Table IV)

**Table IV:** Inter-modality correlation between radiographs and CT for each observer

	**Hip-Knee Angle**			**Sagittal Femoral Angle**			**Sagittal Tibial Angle**		
	**Maximum difference**	**Mean difference**	SD*	**Maximum difference**	**Mean difference**	SD*	**Maximum difference**	**Mean difference**	SD*
Observer 1	6°	1.37°	1.11	8.5°	1.79°	1.49	4.4°	1.45°	1.02
Observer 2	5.4°	1.09°	0.99	12.85°	2.63°	2.62	3.55°	1.3°	0.97

^*^Standard Deviation

**Fig. 4:**
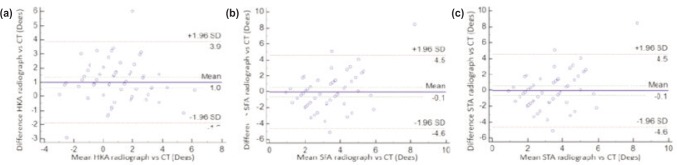
Relationship between difference and mean values of alignment as measured via radiographs and CT by the first observer for (a) Hip-Knee Angle, (b) Sagittal Femoral Angle and (c) Sagittal Tibial Angle.

**Fig. 5:**
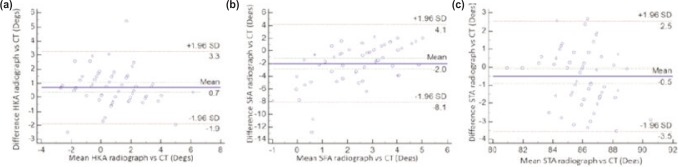
Relationship between difference and mean values of alignment as measured via radiographs and CT by the second observer for (a) Hip-Knee Angle, (b) Sagittal Femoral Angle and (c) Sagittal Tibial Angle.

Graphical representations to indicate agreement between modalities for both observers are provided in Figures 4 and 5.

## Discussion

In this study, we found good to excellent intra- and inter-observer correlation when measuring for HKA, and to a lesser extent, STA. More importantly, these alignment values also showed reliable inter-modality correlation between observers with better comparisons for HKA over STA when comparing radiographic results to CT. Our results with regards to HKA is in keeping with prior literature comparing long-leg radiographs and CT. Babazadeh *et al*^4^ showed excellent inter-observer correlation of r>0.96 and a mean discrepancy between observers of 0.56°. SFA measurements however showed inferior correlation and reproducibility compared to HKA and STA. This questions the validity and reliability of radiographic SFA evaluations when determining sagittal alignment post total knee replacement overall.

Analysing maximum and mean intra- and inter-observer differences gives an idea of the degree of error in these measurements. This error is least pronounced when measuring for HKA while the largest discrepancies were noted when evaluating SFA radiographically. When assessing for agreement between radiographic to CT alignment data, the inaccuracy of SFA measurements were emphasised. For both observers, the greatest maximal error, greatest mean error and largest standard deviation were all found when comparing SFA data between modalities. Agreement between radiographic HKA and STA measurements versus CT were clinically better.

There is contrasting evidence in the current literature regarding the accuracy of radiographic measurements of limb alignment following total knee replacement surgery. Lower limb flexion and rotation may give rise to inaccurate coronal alignment readings as shown previously by Brouwer^9^ and Radtke^17^. Other authors reported poor correlation with the use of non-standardised alignment measures (using anatomical rather than the mechanical axis) and with the use of short-leg films^7,24^. We used standardised radiographic protocols controlling for rotation and flexion at our unit of which we believe, minimises these potential inaccuracies. The use of long-leg radiographs, as in our study, has been shown by Specogna^6^ to be accurate to within 0.9° for coronal measurements.

The main finding in our study was the poor radiological determination of sagittal femoral component alignment using plain radiographs. Though our study employed the use of 18-inch lateral radiographs, we felt that the effect of anterior femoral bowing was significant in giving rise to poor intra- and inter- observer correlation and agreement. Furthermore, there remained a high variability in the geometry of femoral bowing within any given population resulting in difficulties in establishing clear radiological protocols when analysing sagittal component alignment^25^. In a study by Chung^26^*et al*, they identified femoral sagittal discrepancies between the distal anatomical axes versus true mechanical axes of between 5.6° to 8.5°. They also showed that over 20% of their subjects differed more than 2 degrees from the mean value^26^.

Sagittal tibial component measurements in our study showed less discrepancies between radiographic modalities and better intra-/inter- observer correlation and agreement compared to SFA. This may be secondary to better anatomic relationship between proximal tibial anatomic axis projection and the tibial sagittal mechanical axis. Two separate anatomical studies by Han *et al* and Tsukeoka *et al* have shown the relationship between the anatomic and mechanical axes to be 2.2°±0.9^27–28^. This consistency potentially explains the difference in reliability between femoral and tibial component alignment measurements in the sagittal plane using plain radiographs.

Discrepancies in correlation and agreeability between HKA and SFA measurements in our study possibly stemmed from the availability of distinct reference points. In determining the coronal mechanical axis using long-leg radiographs, the centre of the femoral head to the centre of the ankle was often clearly demarcated for measuring alignment. In contrast, the lack of image quality often accompanying cross-table lateral films^29^ makes estimating the femoral head centre difficult and may therefore, reduce accuracy and reproducibility when determining the sagittal femoral mechanical axis. CT imaging would therefore be important in improving the sensitivity and specificity of sagittal femoral component position measurements.

There were several limitations to our study. We took CT data as the standard to compare our inter-modality results while in actual fact, this did not prove that CT was absolutely accurate^30^. Our methods in standardising lateral radiographs could have been improved by employing longer films (including cross-table laterals) and more complex limb positioning devices that allowed for a true assessment of the sagittal mechanical axes. There remains potential discrepancies between data obtained by weight-bearing anterior-posterior four foot films compared to non-weight-bearing CT results.

The purpose of this study was to determine whether conventional lower limb CT imaging following TKA to assess multiplanar knee alignment could be replaced with a coronal and sagittal plain radiography protocol and limited CT imaging of the knee for rotational component positioning. We found that long-leg radiographs remain an excellent tool in determining overall coronal component alignment and to a lesser degree, tibial component sagittal alignment. Radiographs remain a practical measurement method in terms of ease of access coupled with improved cost-effectiveness. However, we reject the study hypothesis that plain radiography is equivalent to CT imaging in accurately determining sagittal plane alignment of the femoral component following TKA. As such, we believe that plain radiography is inferior to CT in assessing postoperative biplanar knee alignment, and CT imaging remains the optimal investigation for precise multiplanar knee alignment.

Further studies looking at the effect of limb rotation on biplanar component alignment measurements using radiographs and CT imaging may improve our understanding regarding the merits of these radiographic modalities following total knee arthroplasty.
